# Identification and Functional Characterization of Cardiac Pacemaker Cells in Zebrafish

**DOI:** 10.1371/journal.pone.0047644

**Published:** 2012-10-16

**Authors:** Federico Tessadori, Jan Hendrik van Weerd, Silja B. Burkhard, Arie O. Verkerk, Emma de Pater, Bastiaan J. Boukens, Aryan Vink, Vincent M. Christoffels, Jeroen Bakkers

**Affiliations:** 1 Hubrecht Institute-KNAW, University Medical Centre Utrecht, Utrecht, The Netherlands; 2 Department of Anatomy, Embryology and Physiology, Heart Failure Research Center, Academic Medical Center, University of Amsterdam, Amsterdam, The Netherlands; 3 Department of Pathology, University Medical Center Utrecht, Utrecht, The Netherlands; 4 Interuniversity Cardiology Institute of the Netherlands, Utrecht, The Netherlands; University of Milan, Italy

## Abstract

In the mammalian heart a conduction system of nodes and conducting cells generates and transduces the electrical signals evoking myocardial contractions. Specialized pacemaker cells initiating and controlling cardiac contraction rhythmicity are localized in an anatomically identifiable structure of myocardial origin, the sinus node. We previously showed that in mammalian embryos sinus node cells originate from cardiac progenitors expressing the transcription factors T-box transcription factor 3 (Tbx3) and Islet-1 (Isl1). Although cardiac development and function are strikingly conserved amongst animal classes, in lower vertebrates neither structural nor molecular distinguishable components of a conduction system have been identified, questioning its evolutionary origin. Here we show that zebrafish embryos lacking the LIM/homeodomain-containing transcription factor Isl1 display heart rate defects related to pacemaker dysfunction. Moreover, 3D reconstructions of gene expression patterns in the embryonic and adult zebrafish heart led us to uncover a previously unidentified, Isl1-positive and Tbx2b-positive region in the myocardium at the junction of the sinus venosus and atrium. Through their long interconnecting cellular protrusions the identified Isl1-positive cells form a ring-shaped structure. In vivo labeling of the Isl1-positive cells by transgenic technology allowed their isolation and electrophysiological characterization, revealing their unique pacemaker activity. In conclusion we demonstrate that Isl1-expressing cells, organized as a ring-shaped structure around the venous pole, hold the pacemaker function in the adult zebrafish heart. We have thereby identified an evolutionary conserved, structural and molecular distinguishable component of the cardiac conduction system in a lower vertebrate.

## Introduction

The cardiac conduction system comprises several components, amongst which the sinus node, the site of electrical impulse generation, and the Purkinje fibers transducing the impulse rapidly through the myocardium [1]. The sinus node harbors specialized pacemaker cells which, due to regular and spontaneous membrane depolarization, generate the electrical signal necessary to induce cardiomyocyte contractions [2]. Although the sinus node was described histologically and functionally more than a century ago [3], the molecular regulators required for pacemaker cell differentiation and function are not fully understood. Nonetheless, several recent developments have provided new insights. These include identification of the embryonic origins of the sinus- and atrioventricular nodes [4], [5] and of several transcriptional regulators involved in their development (reviewed in [1]). A major advance for the field was the identification of T-box transcription factor 3 (Tbx3) in pacemaker cells, and the subsequent demonstration that it is required for sinus- and atrioventricular node development and postnatal homeostasis [6], [7]. Other transcriptional regulators that have been identified for their role in sinus node development are Nkx2.5, Tbx5, Pitx2 and Shox2 [8]–[13]. The molecular signature of the mouse sinus node primordium has been confirmed in human embryonic hearts, indicating evolutionary conservation of the developmental mechanism [14].

The LIM domain transcription factor Isl1 is expressed in the mammalian cardiac progenitor cells [15], [16]. Isl1 expression gradually decreases during differentiation and is eventually lost in mature cardiomyocytes [15], [17], [18], except for myocytes pertaining to the sinus node [19], [20]. Due to structural heart defects and early lethality of mouse embryos deficient for Isl1, its putative role in the developing and mature sinus node has remained elusive.

Although the presence of specialized conduction system components in the heart of lower vertebrates has been suggested by functional analysis [21]–[23], their identification has remained elusive due to the lack of morphologically distinctive structures and molecular markers.

Our research presented in this manuscript resolved this issue by describing the first molecular and structural identification of specialized cardiac pacemaker cells in the embryonic and adult zebrafish heart, utilizing a combination of *in vivo* microscopic examination, 3D gene expression pattern reconstruction, reporter transgenics and *ex vivo* electrophysiology. Our findings establish that Islet-1 is required for pacemaker activity in the embryonic heart and that Islet-1 marks the pacemaker cells in the adult heart, which represents a previously unappreciated role for Isl1 in the cardiac conduction system.

## Results and Discussion

### Cardiac pacemaker activity is affected in Islet-1 mutant hearts

**Figure 1:**
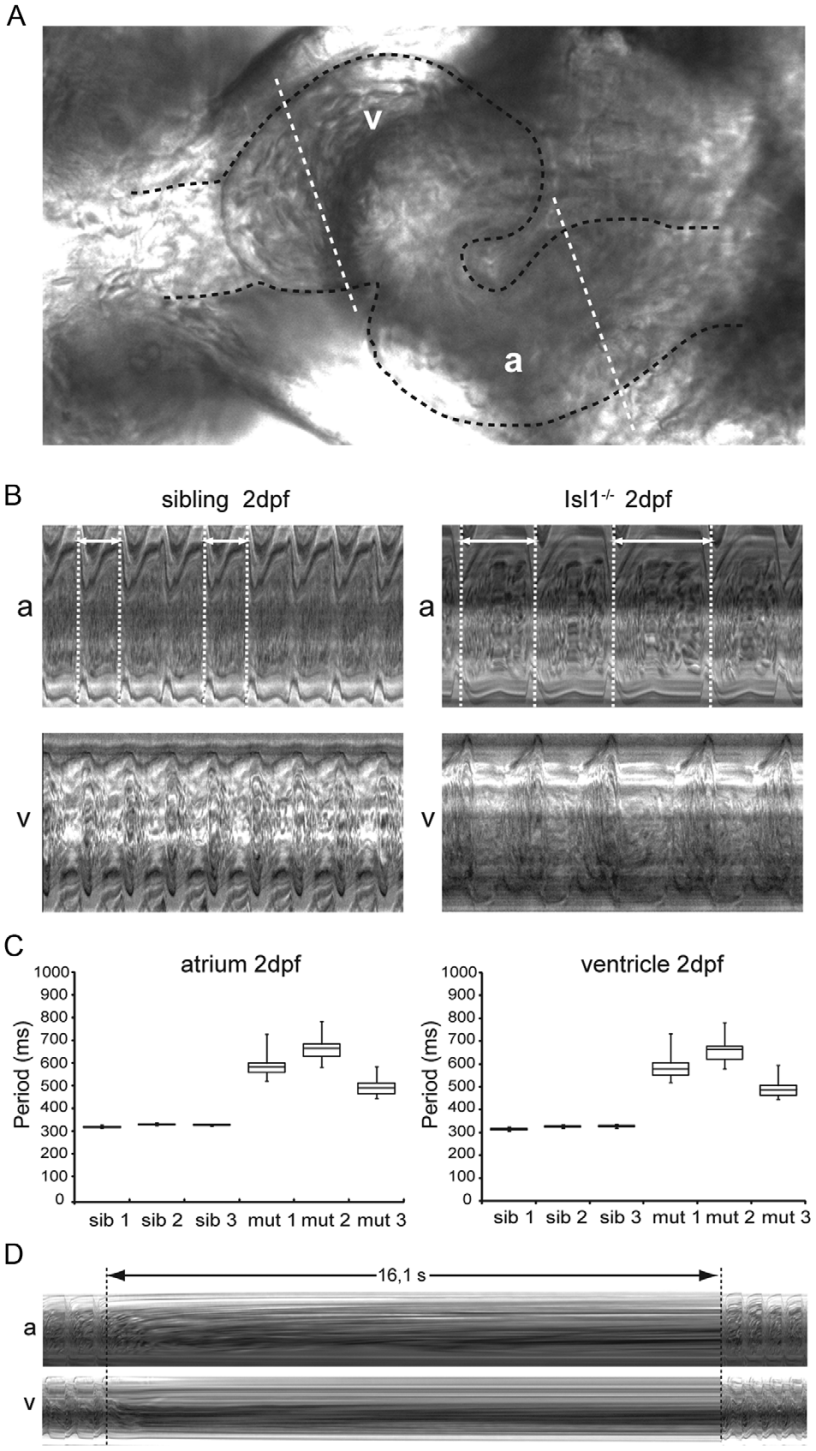
Characterization of the embryonic Isl1^−/−^ cardiac phenotype *in vivo*. (**A**) Zebrafish embryonic heart at 2 dpf. The embryonic heart is highlighted in black dotted contour; white dotted lines through the atrium (A) and the ventricle (V) are placed at kymograph positions. (**B**) Atrial (A) and ventricular (V) kymographs from 2 dpf embryonic hearts spanning approximately 2.8 s. Note the much longer period of the Isl^−/−^ heart when compared to the sibling and the irregularity of the period (double arrow and white dotted vertical lines). Movies are available as Movies S1 and S2, respectively. (**C**) Box-whisker plots representation of 20 successive heartbeats of 2 dpf Isl1^−/−^ and sibling embryos. (**D**) Kymograph recorded at 3 dpf covering a period of about 16 s of absent heart contractions. For all panels a: atrium; v: ventricle.

Zebrafish, embryos lacking functional Isl1 protein are immobile and display reduced heart rate (bradycardia) at 2 days post fertilization (dpf) [24]. Unlike the mouse *Isl1* mutant hearts that fail to loop and lack recognizable chambers [15], zebrafish *isl1* mutant hearts loop normally and are morphologically indistinguishable from their wild-type siblings at 2 days post fertilization (dpf), allowing their functional characterization. To identify the primary defect responsible for the previously reported bradycardia phenotype we further investigated the heart rhythm in *isl1* mutant embryos by high-speed video imaging combined with functional image analysis. Both the *isl1* mutant and wild-type sibling embryos showed initiation of contraction in the inflow region (venous pole) continuing in the atrium, followed by rapid contraction of the ventricle (Movies S1 and S2). To quantify heart rhythm, we drew kymographs for atrium and ventricle of sibling and mutant hearts using a 1 pixel-thick collection of lines positioned perpendicularly to the blood flow (Fig. 1A, white dotted lines). The resulting kymographs (Fig.1B) readily confirmed the bradycardia of the *isl1* mutant, as the distance between two dotted lines encompassing a full cardiac cycle (Fig.1B, white double arrows) is much longer in the mutant than in the sibling. Moreover, the kymograph-based quantification of the cardiac cycle (interval necessary for one complete contraction) illustrates the slow and irregular heart rhythm in *isl1* mutant embryos (Fig. 1C). Cardiac cycles of the imaged *isl1* mutants oscillated between about 450 ms and 800 ms, showing not only high variability within a single embryo (Fig. 1C, box-whisker plots for mutants; maximal and minimal values vary of about 200 ms for all mutants analyzed), but also between different individuals (Fig.1C, mutants 1,2 and 3 display average cardiac cycle period times of 586 ms, 661 ms and 494 ms respectively over the recording time). In comparison, siblings displayed a maximal variation in cardiac cycle measured at the atrium of about 20 ms and average cardiac cycle periods of 325 ms (Fig. 1C, Movie S1). We never observed uncoupling of the atrial and ventricular contraction, fibrillation or atrioventricular block, as every atrial contraction was followed by a ventricular contraction (Fig. 1B; Movie S2).

As development proceeded, the severity of the heart beat phenotype increased. We frequently observed a sinus block in *isl1* mutant hearts at 3–4 dpf resulting in the absence of atrial and ventricular contraction for 10–20 seconds (Fig. 1D). Altogether, the combination of phenotypes displayed by the *isl1* mutant is compatible with defective initiation of contraction, suggesting faulty pacemaker activity.

### Isl1 is expressed at the putative sinoatrial boundary of the embryonic and adult heart

In mammals, including humans, the primary pacemaker function is held by the sinus node, which resides at the junction of the superior caval vein and right atrium [25]–[28]. For example, in mouse, the right sinus venosus was shown to form the sinus node, which includes the venous lining of the right venous valve [4], [13]. Isl1-expressing cells are found in the developing and mature mammalian sinus node [14], [19], [20].

**Figure 2:**
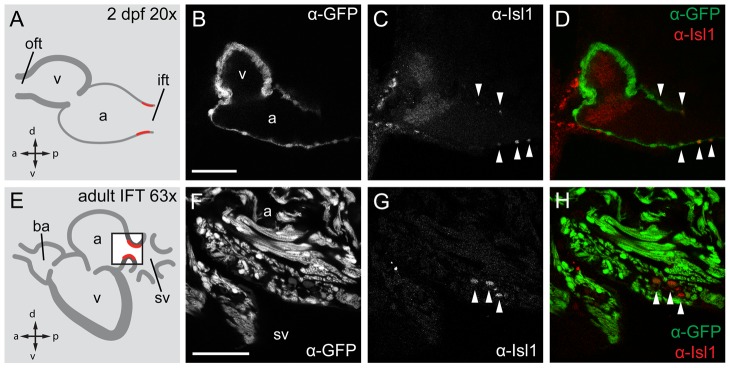
Isl1 expression in the embryonic and adult zebrafish heart. Single confocal scans of a fluorescent antibody labeling of Isl1 and eGFP in embryonic (2 dpf) (**A–D**) and adult (**E–H**) zebrafish expressing *Tg(myl7:eGFP)* in all cardiomyocytes. GFP^+^ cardiomyocytes are displayed in grey (B, F) and in green in (D, H). Isl1 is shown in grey (C, G) and in red (D, H). Arrowheads indicate Isl^+^/GFP^+^ cells. Illustrations of a lateral view of a 2 dpf (A) and adult (E) zebrafish heart indicate the location of Isl1^+^ cells (red). The box in panel E represents the area shown in (F–H). (**B–D**) Fluorescent immunolabeling of Isl1 and eGFP in a 2 dpf embryo (sagittal section 100 µm). At this time point Isl1^+^/GFP^+^ cells were only found in the IFT of the heart. (**F–H**) Fluorescent immunolabeling of Isl1 and eGFP in an adult zebrafish heart (sagittal section 100 µm). Isl1^+^/GFP^+^ cells are located at the junction of the sinus venosus and atrium in the inflow region of the heart (arrowheads). Isl1^+^ cells showed low expression of *myl7*. v, ventricle; a, atrium; oft, outflow tract; ift, inflow tract; ba, bulbus arteriosus; sv, sinus venosus; a, anterior; p, posterior; d, dorsal; v, ventral. Scale bars represent 50 µm.

**Figure 3:**
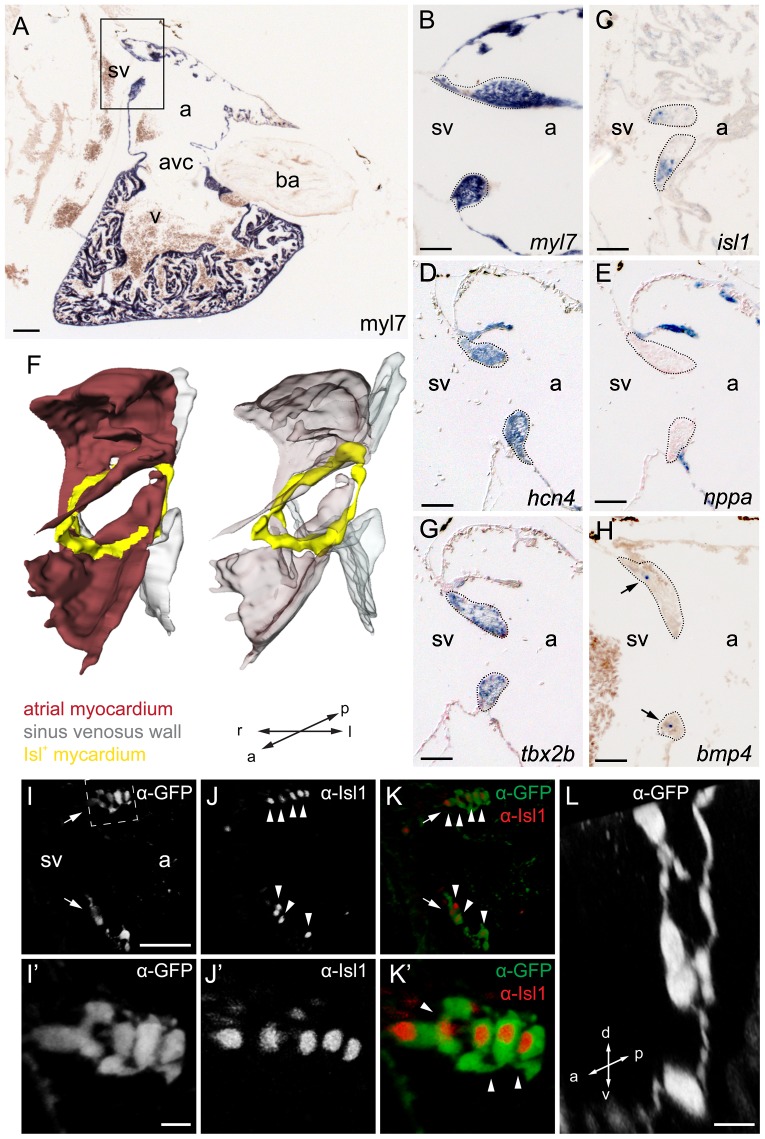
Molecular characterization of the adult isl1 expression domain. (**A–E,G,H**) Section in-situ hybridizations on adult wild-type zebrafish hearts. Probes are indicated in the panels. (A) 4-chamber view of a sagittal section through zebrafish heart labeled with the myocardial marker myl7. The box indicates the region shown enlarged in panels B–E,G,H. Demarcated areas (C) indicate isl1 expression in the *tbx2b+ hcn4+ nppa-* myocardium at the base of the valves surrounding the sinoatrial junction. BMP4 signal is pointed at in the valves by arrowheads in (H). (**F**) 3D reconstruction of the sinoatrial junction. *isl1* (yellow) is expressed around the entire sinoatrial junction, forming a ring-like structure. (**I–L**) Reconstruction of a confocal scan through a sagittal section of the inflow region of adult Tg(isl1BAC:GalFF; UAS:GFP) transgenic zebrafish heart. Fluorescent antibody labeling for GFP (shown in grey (I, I', L) or green (K, K')) and for Isl1 (shown in grey (J, J') or red (K, K')). GFP+/Isl1+ cells were found in bilateral populations at the sinoatrial junction (arrows). (I'–K') Enlargement of the GFP+/Isl1+ cells at the dorsal rim of the inflow tract (indicated with dashed box in (I)). Nuclei of GFP+ cells were positive for Isl1 (arrowheads). (L) Enlargement of GFP+ cells (transverse section) shows a string of contiguous cells. a, atrium; avc, atrioventricular canal; ba, bulbus arteriosus; sv, sinus venosus; v, ventricle; l, left; r, right; a, anterior; p, posterior; d, dorsal; v, ventral. Scale bars represent 50 µm (A–G, I–K) or 10 µm (I'–K',L).

We hypothesized that Isl1 expression marks the pacemaker tissue in the zebrafish heart, as no molecular markers for the zebrafish sinus node or the pacemaker cells within it have been identified to date. Using an antibody recognizing both zebrafish Isl1 and Isl2 [29] we observed few Isl-positive cells in both the dorsal and ventral regions of the proposed sinoatrial junction at 2 dpf (Fig. 2A, B, C, D), consistent with the proposed pacemaker function at the sinoatrial junction in the zebrafish heart [21]. The Isl-expressing cells expressed *Tg(myl7:eGFP)*, which marks cardiomyoctes. Isl expression at the sinoatrial junction was detected continuously during development and was maintained in the same region in the adult zebrafish heart (Fig. 2E, F, G, H). This indicates that Isl expression in the zebrafish proposed sinoatrial junction is constant between embryonic stages and adulthood. We continued by molecular characterization of the proposed sinoatrial junction region in the adult heart using in situ hybridization (ISH). Contrary to what has been observed in mammals, the zebrafish sinus venosus did not express the myocardial marker *myl7*. Consequently, the upstream border of myocardium muscle was at the venous valves and atrium (Fig. 3A, B). We observed that *isl1* expression was confined to the myocardium located at the base of the venous valves (Fig. 3C). The zebrafish ortologue of *hcn4* encodes a member of the family of ion channels responsible for the hyperpolarization-activated current, *I*_f_, in pacemaker cells. Its expression is enriched in pacemaker tissue of mammalian hearts [28], [30]–[33]. In the zebrafish adult heart *hcn4* had a broader expression pattern than *isl1* (Fig. 3D), similar to observations made in the mammalian embryonic heart [13], [14]. Expression of the two genes overlapped at the sinoatrial junction (Fig. 3D). A 3D reconstruction based on a series of ISH on sagittal adult heart sections revealed that *isl1* expression is confined to a ring-like structure within the myocardial tissue at the base of the venous valves, around the proposed sinoatrial junction (Fig. 3F). In the mammalian heart expression of *Nppa* is specific for the fast conducting working myocardium of the atrium and ventricle but is not expressed in the slow conducting primitive myocardium of the pacemaker tissue at the sinoatrial junction [13]. We observed a very similar mutually exclusive expression pattern of *nppa* and *isl1* at the proposed sinoatrial junction of the zebrafish heart (Fig. 3E). Furthermore, since *bmp4* and *tbx2b* are expressed at the venous pole of the embryonic heart ([24] and Figure S1) and since the bmp4-tbx2b regulatory axis suppresses chamber differentiation and *nppa* expression, we analyzed their expression in the adult heart. Interestingly, expression of *bmp4* and *tbx2b* was maintained in the myocardium of the proposed sinoatrial junction of the adult heart and restricted to the base of the venous valves or the entire venous valve tissue, respectively (Fig. 3G, H). In summary, we identified an *isl1*+cell population in the *hcn4*+*tbx2b*+*nppa*-negative myocardium that is organized in a ring-shape at the proposed sinoatrial junction. Together with the above-described observation that *isl1* mutants display irregular heart rhythms, this suggests that *isl1* expression identifies cardiac pacemaker cells in the zebrafish heart.

Using an Isl1-LacZ knock-in model, Isl1/LacZ activity was observed in the sinus node of the adult mouse heart [20]. Corroborating these findings we detected endogenous Isl1 expressing cells in the adult sinus node of the mouse heart using an anti-Isl antibody (Figure S2). Using a similar approach we also detected Isl1 expressing cells in the sinus node of the adult human heart (Figure S3). Interestingly, we observed that only a sub-population of sinus node cells expresses Isl1, suggesting that the Isl1 expressing cells have different properties compared to the isl1-negative sinus node cells.

### Isl1-expressing cells display electrical pacemaker activity

To functionally characterize the Isl1+ cells in the zebrafish heart, we generated an Isl1-GFP reporter line using the binary Gal4/UAS expression system (Figure S4). To validate the reporter line we confirmed that all GFP+ cells were co-labeled by Isl1 immunostaining (Fig. 3I, J, K' arrowheads). In vibratome sections GFP+ cells visualized by fluorescent antibody labeling were located in bilateral cell populations at the proposed sinoatrial junction (Fig. 3I, J, K, arrows), conform the ISH staining. Neighboring GFP+/Isl1+ cells displayed cytoplasmic protrusions, which may connect them between each other (Fig. 3K', arrowheads). 3D reconstruction of confocal image stacks revealed that Isl1+ pacemaker cells are interspersed with Isl-negative cells. However, they form a coherent structure (Fig. 3L). It is known that isolated groups of cells with residual pacemaker activity, such as remnant embryonic nodal atrioventricular canal myocytes, may cause cardiac arrhythmias (reviewed in [34], [35]). The cell-to-cell interconnection could therefore be an essential feature for proper pacemaker function, likely coordinating a synchronous activation of the myocardium. Moreover, the expression of myosin is low in Isl+ cells, which is supportive for a primitive myocardial identity of these cells, typical for pacemaker cells (Figure S5) [27], [36], [37].

**Figure 4:**
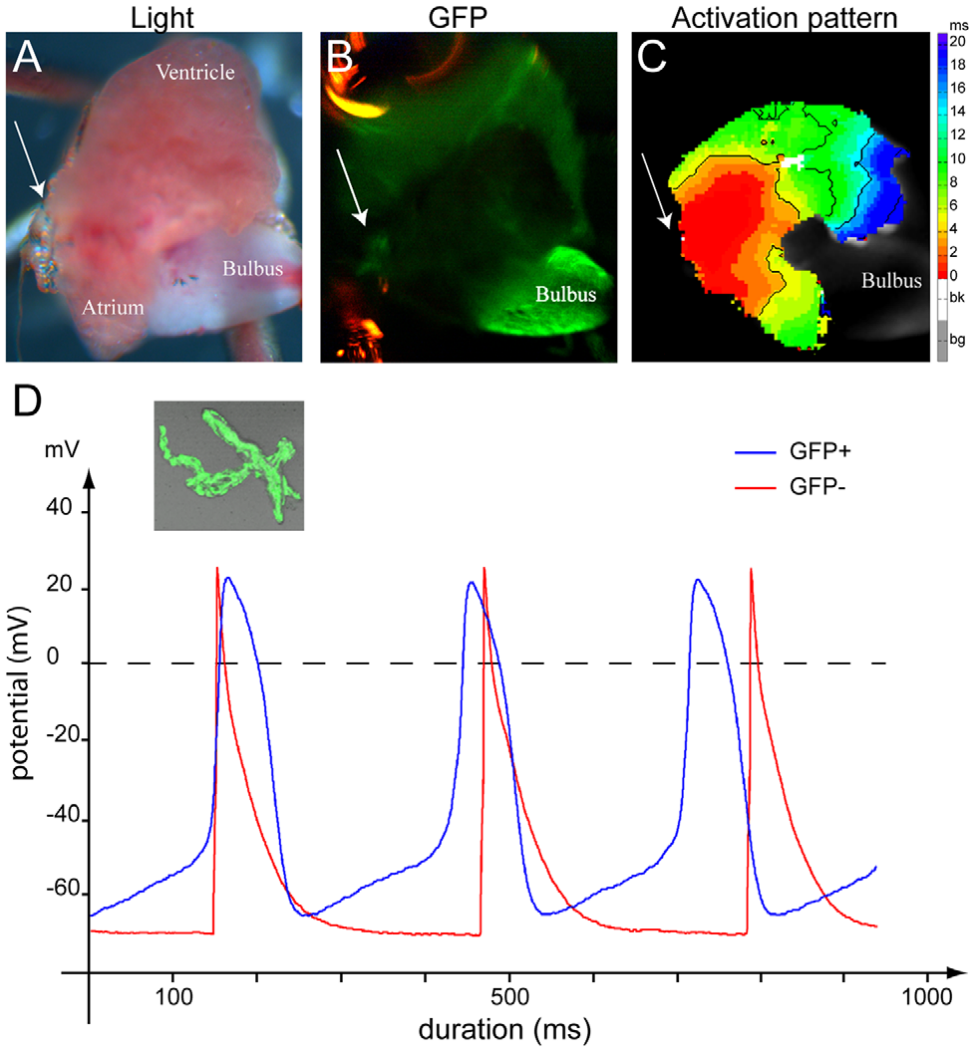
Isl1 cells have pacemaker activity. (**A–C**) Optical mapping on an explanted, contracting adult zebrafish tg(isl1BAC:GalFF; UAS:GFP) heart. Arrow indicates the sinus venosus in all panels. (A) Explanted adult zebrafish heart. (B) GFP-fluorescent cells reporting Isl1 expression are situated at the sinus venosus. (C) The activation pattern measured by di-4-ANEPPS fluorescence shows that the GFP+ myocytes are situated in the area of earliest activation. (**D**) Typical action potentials of freshly isolated GFP^+^ and GFP^−^ myocytes. The GFP^−^ cell was stimulated at 3 Hz. The inset displays a representative example of a GFP+ myocyte.

To elucidate whether the GFP+ cells localize to the region in which the electrical activation is initiated, we performed optical mapping of epicardial activation patterns on adult zebrafish hearts. First moment of atrial activation corresponded with the localization of the GFP+ cells (Fig. 4A, B, C), which is compatible with pacemaker function for *isl1* expressing cells.

To unequivocally discriminate whether indeed *isl1* expression marks cells with pacemaker activity, dissociated single GFP+ and GFP- cells from micro-dissected sinoatrial tissue were patch-clamped (Fig.4D). All measured GFP+ cells (n = 6) were spontaneously active, while all measured GFP- cells (n = 8) were typically quiescent. In GFP- cells, action potentials could be elicited by current pulses through the patch pipette. Figure 4D shows typical spontaneous action potentials of a GFP+ cell as well as typical action potentials recorded from a GFP- cell that was stimulated at 3 Hz, i.e. with a cycle length similar close to that of the GFP+ cell (Table 1). All parameters, except for action potential duration at 90% repolarization (APD_90_), differed significantly between both cell types (Table 1). GFP- cells had a stable resting membrane potential of -79.0±1.6 mV, while GFP+ cells showed a spontaneous diastolic depolarization rate (64±17 mV/s) with a maximum diastolic potential of −65.0±3.0 mV. In GFP+ cells, this diastolic depolarization resulted in pacemaker activity with an intrinsic cycle length of 378±58 ms. In GFP+ cells, the maximum upstroke velocity was typically low (7.4±2.6 V/s) as opposed to GFP- cells (112±14 V/s). In both cell types, action potentials overshot the zero potential value, but the action potential amplitude was higher in GFP- cells. Action potentials of GFP- cells repolarized earlier and faster, resulting in shorter APD_20_ and APD_50_. Thus, the results obtained on the optical mapping and patch-clamp experiments revealed that while GFP- cells display characteristics specific to cardiac chamber myocytes, pacemaker activity resides in Isl+/GFP+ cells.

**Table 1:** Action potential characteristics of single GFP^+^ and GFP^−^ cells.

	GFP^+^ cells (n = 6)	GFP^−^ cells (n = 8)
cycle length (ms)	377±58	–
MDP (mV)	−65.0±3.0	−79.0±1.6*
DDR_50_ (mV/s)	63.4±16.8	–
dV/dt_max_ (V/s)	7.3±2.6	112±14*
APA (mV)	78.5±7.5	98.2±3.5*
APD_20_ (ms)	48.1±6.5	12.5±2.6*
APD_50_ (ms)	71.1±8.6	35.8±2.8*
APD_90_ (ms)	100.0±13.3	82.5±10.7

Data are mean±SEM; n = number of cells, MDP = maximal diastolic potential, DDR_50_ = diastolic depolarization rate over the 50-ms time interval starting at MDP+1 mV, dV/dt_max_ = maximal upstroke velocity, APA = action potential amplitude, APD_20_, APD_50_, and APD_90_ = action potential duration at 20, 50, and 90% repolarization. *p<0.05.

## Conclusions

We present here the molecular and functional identification of cardiac pacemaker cells in the embryonic and adult zebrafish heart. The embryonic cardiac expression pattern (Fig.2) and knockout phenotype of Isl1 in zebrafish (Fig.1) hinted at a role for this gene in pacemaker function. Indeed, analysis of 3D reconstructions of expression pattern and reporter transgenics in adult fish showed a previously unidentified ring-shaped region of Isl1 expression within the myocardium at the proposed sinoatrial junction of the mature zebrafish heart (Fig.3). Optical mapping of the activation sequence and electrophysiological characterization of single Isl1+ myocytes demonstrated the presence of pacemaker activity in the Isl1-expressing cells (Fig. 4). Altogether, our data allow us to establish that (1) Isl1 is the first identified molecular marker for pacemaker cells in the zebrafish heart, (2) the functional pacemaker of the adult zebrafish heart is organized as a ring around the venous pole and (3) expression of Isl1 in the pacemaker cells of the adult heart is conserved from fish to human.

Cells pertaining to the primary pacemaker structure in zebrafish and mammals share a number of molecular markers, relative position in the heart, and functional identity. However, while in zebrafish the Isl1-expressing pacemaker cells are few and organized in a ring-shaped structure of interconnected cells in the venous valves, the mammalian sinus node is a more compact, spindle-shaped (also referred to as comma-shaped) clearly demarcated structure. Although at this stage we do not know whether the ring-shaped pacemaker can be extended to other lower vertebrates, the diverging and unpronounced structure of the zebrafish sinus node and the absence of molecular markers until now may explain why a pacemaker structure in lower vertebrates has not previously been identified.

Finally, the ‘primitive’ myocardial identity of Isl1+ cells in adult zebrafish is intriguingly accompanied by a capacity to spontaneously depolarize, which is absent in working cardiomyocytes. Future work focusing on Isl1 and its gene targets will help to elucidate whether Isl1 expression in the pacemaker cells is necessary to maintain the primitive myocardial fate of these cells, similarly to its suggested role in cardiac progenitor cells [15], [17], [18], [38]. Alternatively, Isl1 could be required to drive a pacemaker gene program, in a similar fashion to what was shown for Tbx3 [7].

## Materials and Methods

### Ethics statement

All animal and human work conformed to ethical guidelines and was approved by the relevant

local animal ethics committees.

### Animal work

Animal Experimental Committee

Secretariaat DEC/KNAW

Koninklijke Nederlandse Akademie voor Wetenschappen

Postbus 19121

1000 GC Amsterdam

### Human Tissue Samples

The study met the criteria of the code of proper use human tissue that is used in the Netherlands for the use of human tissue. The study was approved by the scientific advisory board of the biobank of the University Medical Center Utrecht (RP2012-03).

### Zebrafish lines

Fish used in this study were kept in standard conditions as previously described [39]. The *Tg(myl7:gfp)* and isl1 mutant line *isl1K88X (isl1^SA0029^)* were described previously [24], [40]. Generation of the *Tg(Isl1BAC:GalFF; UAS:GFP)* is described in more detail below. All animal and human work conformed to ethical guidelines and was approved by the relevant local animal ethics committees.

### Generation of the *tg(isl1BAC:Gal4ff)* transgenic line

Recombineering of BAC clone CH211-219F7 was performed following the manufacturer's protocol with minor modifications, as described in [41]. Primers used were: isl1_Gal4FF_F

5′-gggccttctgtccggttttaaaagtggacctaacaccgccttactttcttaccATGAAGCTACTGTCTTCTATCGAAC-3′

and isl1_KanR_R

5′-aaataaacaataaagcttaacttacttttcggtggatcccccatgtctccTCAGAAGAACTCGTCAAGAAGGCG-3′.

BAC DNA was injected at 300 ng/µl in the presence of 0.75U PI-SceI meganuclease (New England Biolabs) into *Tg(UAS:GFP)* embryos [42]. Healthy embryos displaying robust isl1-specific fluorescence were grown to adulthood. *Tg(Isl1BAC:GalFF; UAS:RFP)* were obtained by outcross to a *tg(UAS:RFP)* line [42].

### High-speed imaging and analysis

2 Dpf and 4 dpf *isl1K88X* mutant and sibling embryos were mounted in 0.25% agarose (Life Technologies BV) prepared in E3 medium embryonic medium with 16 mg/ml 3-amino benzoic acid ethylester. Embryonic hearts were imaged with a Hamamatsu C9300-221 high speed CCD camera (Hamamatsu Photonics, Hamamatsu City, Japan) at 150 fps mounted on a Leica AF7000 microscope (Leica Microsystems GmbH, Wetzlar, Germany) in a controlled temperature chamber (28.5°C) using Hokawo 2.1 imaging software (Hamamatsu Photonics GmbH, Herrsching am Ammersee, Germany). Image analysis was carried out with ImageJ (http://rsbweb.nih.gov/ij/). Statistical analysis and drawing of the box-whisker plot were carried out in Excel 2007 (Microsoft, Redmond, WA, USA).

### In situ hybridisation and immunohistochemistry

ISH on embryos was carried out as previously described [39]. ISH on adult heart tissue was carried out as previously described [43] with minor modifications. 3D reconstructions of serial ISH-labeled sections were performed as described in [44]. Immunohistochemistry was carried out as previously described [24]. Embryos and adult hearts for immunocytochemistry were fixed in 2% paraformaldehyde, embedded in 3% agarose/1% gelatine and sectioned at 100 µm thickness. The primary antibodies used were mouse anti-Isl1 (Developmental Studies Hybridoma Bank, Iowa City, IA, USA, clone 39.4D5, 1∶100), mouse-anti-tropomyosin (Sigma-Aldrich, Zwijndrecht, the Netherlands, Cat. No. T9283, 1∶100), rabbit anti-GFP (Torrey Pines Biolabs Inc., Secaucus, NJ, USA, Cat. No. TP401, 1∶200) and rabbit-anti-DsRed (Clontech Laboratories Inc., Mountain View, CA, USA, Cat No. 632496, 1∶100).

### Optical Mapping

Hearts from adult *tg(isl1:GalFF; UAS:GFP)* fish were excised and incubated in 10 ml Ringer's solution (composition in mM: NaCl 115, Tris 5, NaH_2_PO_4_ 1, KCl 2.5, MgSO_4_ 1, CaCl_2_ 1.5, glucose 5, pH adjusted to 7.2 with HCl) containing 15 µM Di-4 ANEPPS for 5 minutes and placed in an inverted microscope. Excitation light was provided by a 5-Watt power LED (filtered 510±20 nm). Fluorescence (filtered>610 nm) was transmitted through a tandem lens system on CMOS sensor (100×100 elements, MICAM Ultima, SciMedia, Costa Mesa, CA, USA). Activation patterns were measured during the sinus rhythm. Optical action potentials were then analyzed with custom-made software.

### Single cell preparation

Single cells were isolated from the sinoatrial node and atria as described previously [45]. Sinoatrial node regions were excised from 55 adult *tg(Isl1BAC:GalFF; UAS:GFP)* zebrafishes and stored in Tyrode's solution at RT, containing (in mM): NaCl 140, KCl 5.4, CaCl_2_ 1.8, MgCl_2_ 1.0, glucose 5.5, and HEPES 5.0; pH was set to 7.4 with NaOH. They were then transferred to Ca^2+^-free Tyrode's solution (30°C), i.e., Tyrode's solution with 10 µM CaCl_2_, which was refreshed two times before the addition liberase IV (0.25–0.29 U/ml; Roche, Indianapolis, IN, USA) and elastase (2.4–0.7 U/mL; Serva, Heidelberg, Germany) for 12–15 min. The final 6 min also contained pronase E (0.92 U/mL; Serva). During the incubation period, the tissue was triturated through a pipette (tip diameter: 2.0 mm). The dissociation was stopped by transferring the strips into a modified Kraft-Brühe solution (30°C) containing (in mM KCl 85, K_2_HPO_4_ 30, MgSO_4_ 5.0, glucose 20, pyruvic acid 5.0, creatine 5.0, taurine 30, β-hydroxybutyric acid 5.0, succinic acid 5.0, BSA 1%, Na_2_ATP 2.0; pH was set to 6.9 with KOH. The tissue was triturated (pipette tip diameter: 0.8 mm) in Kraft-Brühe solution (30°C) for 4 min to obtain single cells, which were stored at RT for 30 min in modified Kraft-Brühe solution before patch-clamping. Cells were allowed to adhere for 5 min after which superfusion with Tyrode's solution (28.5±0.2°C) was started.

### Patch clamp experiments

Action potentials were recorded by the amphotericin-perforated patch-clamp technique using an Axopatch 200B amplifier (Molecular Devices, Sunnyvale, CA, USA). Action potentials of GFP+ cells were low-pass filtered (cut-off frequency 5 kHz) and digitized at 10 kHz; action potentials of GFP- cells at 5 kHz and 20 kHz, respectively. Potentials were corrected for the estimated liquid junction potential [46]. Data acquisition and analysis were accomplished using custom software. Patch pipettes (borosilicate glass; resistance 3–4 MΩ) were heat polished and filled with pipette solution containing (in mM): K-gluc 125, KCl 20, NaCl 10, amphotericin-B 0.22, and HEPES 10; pH was set to 7.2 with KOH. Action potentials in GFP-negative cells were elicited at 3 Hz by 3-ms, ≈1.2x threshold current pulses through the patch pipette. Parameter values obtained from 10 consecutive action potentials were averaged.

## Supporting Information

Figure S1 **Expression of** ***tbx2b*** **at the venous pole in embryonic heart.** Expression patterns by mRNA in situ hybridization of *tbx2b* and *nppa* in 2 dpf embryos. Expression of *tbx2b* at the venous pole (blue staining indicated with arrowheads) does not overlap with *nppa* expression (red staining), which is confined to atrium and ventricle chamber myocardium. Pictures shown are ventral views with anterior to the top.
(TIF)

Figure S2 **Isl1 expression in the sinus node of the adult mouse heart.** **(A)** 4-chamber view of section through adult wild-type mouse heart. Boxed region indicates the region shown enlarged in (B). **(B)** Expression of Isl1, depicted in red, colocalizes with the expression of the sinus node marker Hcn4, depicted in green. Dotted line in (B) demarcates the sinus node. ao, aorta; la, left atrium; lv, left ventricle; ra, right atrium; rv, right ventricle; scv, superior caval vein.
(TIF)

Figure S3 **Immunohistochemical detection of Islet-1 in human cardiomyocytes in the sinoatrial node.** **(A)** Hematoxylin and eosin staining of the sinoatrial node. SN indicates the area of the node showing where the specialized cardiomyocytes are located. MC indicates myocardium adjacent to the node. NA indicates the nodal artery. The boundary between SN and MC is highlighted by the dotted line. Scale bar represents 400 µm. **(B)** Elastic van Giesen stain of a consecutive section of (A) illustrating that the cardiomyocytes are embedded within collagen and elastic tissue. Scale bar represents 400 µm. **(C)** Islet-1 immunostain of sinoatrial node. SN indicates sinoatrial node. MC indicates myocardium adjacent to the node. The boundary between SN and MC is highlighted by the dotted line. Scale bar represents 160 µm. **(D)** Magnification of the boxed region in (C). Islet-1 immunostain with positive brown staining of the nuclei of the cardiomyocytes. On average 5% of the cardiomyocytes in the sinoatrial node revealed a positive signal. Scale bar represents 40 µm. **(E)** Staining is absent in the myocardium next to the sinoatrial node. Scale bar represents 80 µm.
(TIF)

Figure S4 **Generation of a reporter transgenic line for isl1. (A)** An expression cassette containing the GalFF gene [42] and kanamycin resistance gene was inserted by recombineering into BAC CH211-219F7 at the ATG site of the 1st exon of the isl1 gene. The site of recombineering is approximately 40 kb inside the BAC sequence, minimizing any risk of loss of isl1 regulatory sequences. The recombined BAC was then injected in a tg(UAS:GFP) background [1] to obtain the fluorescent Isl1 expression reporter line Tg(Isl1BAC:GalFF; UAS:GFP). **(B)** GFP expression pattern of the Tg(Isl1BAC:GalFF; UAS:GFP) line at 3 dpf. **(C)** Isl1 ISH on WT embryo at 3 dpf. The expression pattern of GFP, reporting for isl1 expression, in the Tg(isl1BAC:GalFF; UAS:GFP) is validated by comparison with the isl1 ISH. Especially visible are the identical expression pattern in the eyes and hindbrain.
(TIF)

Figure S5 **Isl1BAC reporter activity in adult heart.** Confocal images of Tg(isl1BAC:GalFF; UAS:RFP; myl7:eGFP) after immunolabeling with anti-RFP and anti-GFP antibodies **(A)**, or Tg(isl1BAC:GalFF; UAS:GFP) after immunolabeling with anti-GFP and antitropomyosin antibodies **(B)**. Isl1 expressing cells (indicated with arrows) are located at the base of the venous valves and contain much lower levels of myosin light chain or tropomyosin compared to surrounding myocardial cells. Axonal Isl1/GFP+structures are visible at the outer surface of the myocardium. Scale bars represent 50 µm
(TIF)

Movie S1 **Recording of the heart beating of an** ***isl1*** **sibling embryo at 2 dpf.** Ventral view (anterior to the left). Note the speed and regularity of the contractions (also see Fig.1B). Playback speed (150 fps) is set to match recording speed to render natural speed of the embryo's heart beat.
(AVI)

Movie S2 **Recording of the heart beating of an** ***isl1^−/−^*** **embryo at 2 dpf.** Ventral view (anterior to the left). Note the reduced speed of the contractions and their irregularity (also see Fig.1B). Playback speed (150 fps) is set to match recording speed to render natural speed of the embryo's heart beat.
(AVI)
