# Supplementary figures and images for: Identification and Functional Characterization of Cardiac Pacemaker Cells in Zebrafish
Source: PLoS One. 2012 Oct 16;7(10):e47644. doi: 10.1371/journal.pone.0047644 (PMC3473062; doi:10.1371/journal.pone.0047644)

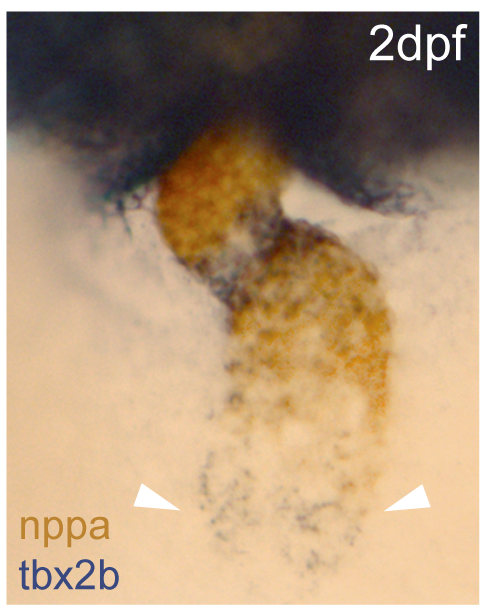

Supplement: Figure S1 — Expression of tbx2b at the venous pole in embryonic heart. Expression patterns by mRNA in situ hybridization of tbx2b and nppa in 2 dpf embryos. Expression of tbx2b at the venous pole (blue staining indicated with arrowheads) does not overlap with nppa expression (red staining), which is confined to atrium and ventricle chamber myocardium. Pictures shown are ventral views with anterior to the top. (TIF) [file pone.0047644.s001.tif]

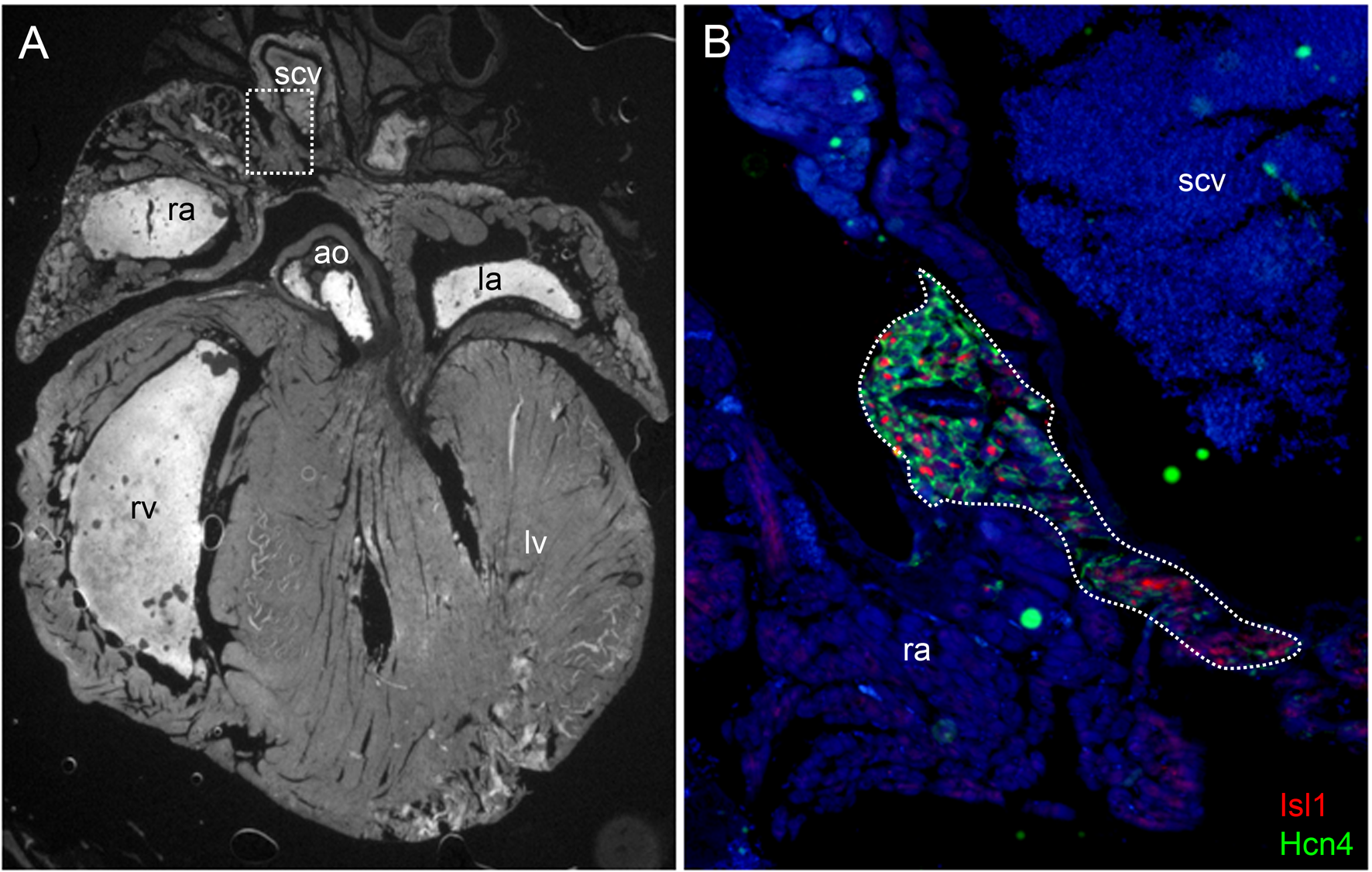

Supplement: Figure S2 — Isl1 expression in the sinus node of the adult mouse heart. (A) 4-chamber view of section through adult wild-type mouse heart. Boxed region indicates the region shown enlarged in (B). (B) Expression of Isl1, depicted in red, colocalizes with the expression of the sinus node marker Hcn4, depicted in green. Dotted line in (B) demarcates the sinus node. ao, aorta; la, left atrium; lv, left ventricle; ra, right atrium; rv, right ventricle; scv, superior caval vein. (TIF) [file pone.0047644.s002.tif]

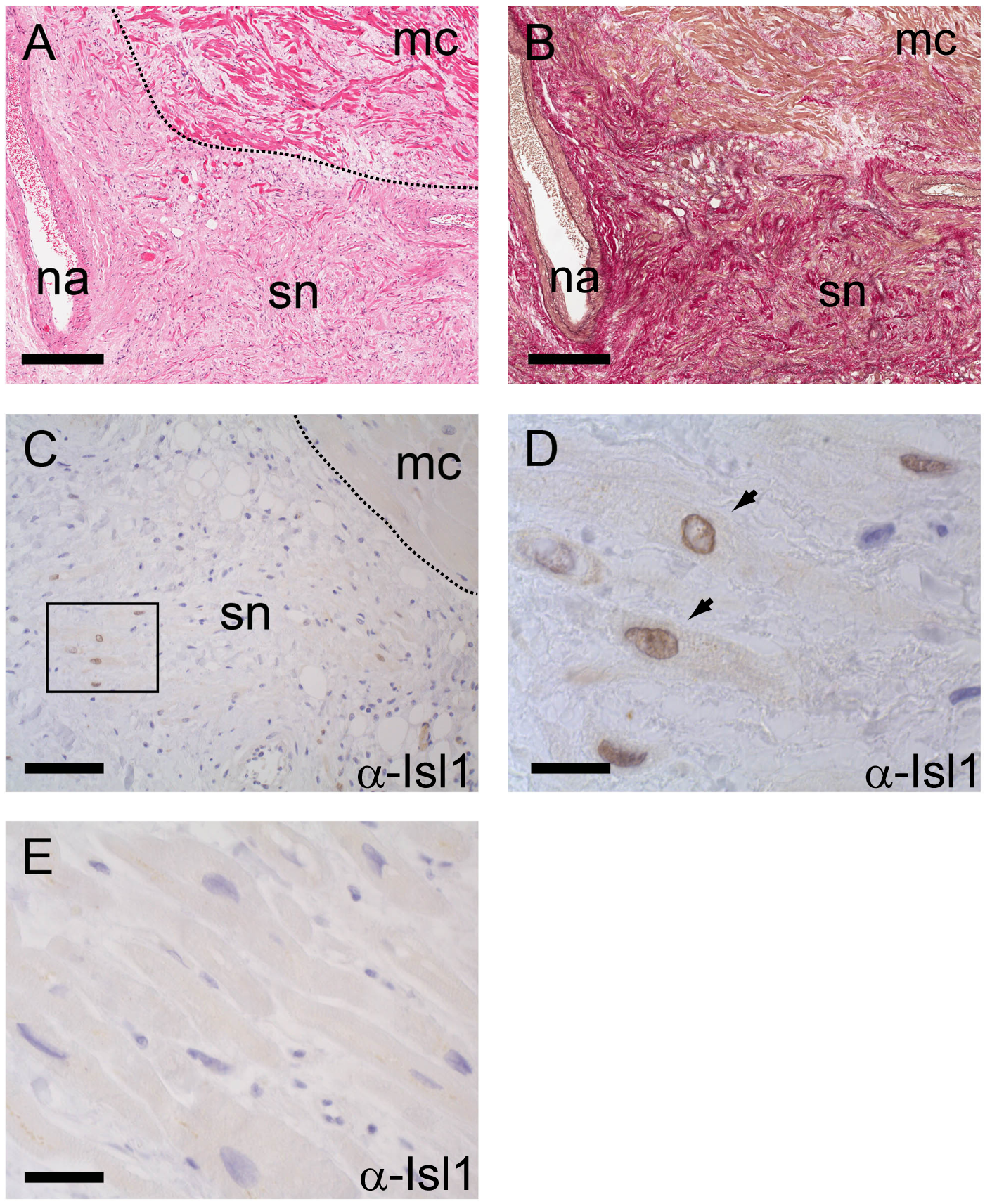

Supplement: Figure S3 — Immunohistochemical detection of Islet-1 in human cardiomyocytes in the sinoatrial node. (A) Hematoxylin and eosin staining of the sinoatrial node. SN indicates the area of the node showing where the specialized cardiomyocytes are located. MC indicates myocardium adjacent to the node. NA indicates the nodal artery. The boundary between SN and MC is highlighted by the dotted line. Scale bar represents 400 µm. (B) Elastic van Giesen stain of a consecutive section of (A) illustrating that the cardiomyocytes are embedded within collagen and elastic tissue. Scale bar represents 400 µm. (C) Islet-1 immunostain of sinoatrial node. SN indicates sinoatrial node. MC indicates myocardium adjacent to the node. The boundary between SN and MC is highlighted by the dotted line. Scale bar represents 160 µm. (D) Magnification of the boxed region in (C). Islet-1 immunostain with positive brown staining of the nuclei of the cardiomyocytes. On average 5% of the cardiomyocytes in the sinoatrial node revealed a positive signal. Scale bar represents 40 µm. (E) Staining is absent in the myocardium next to the sinoatrial node. Scale bar represents 80 µm. (TIF) [file pone.0047644.s003.tif]

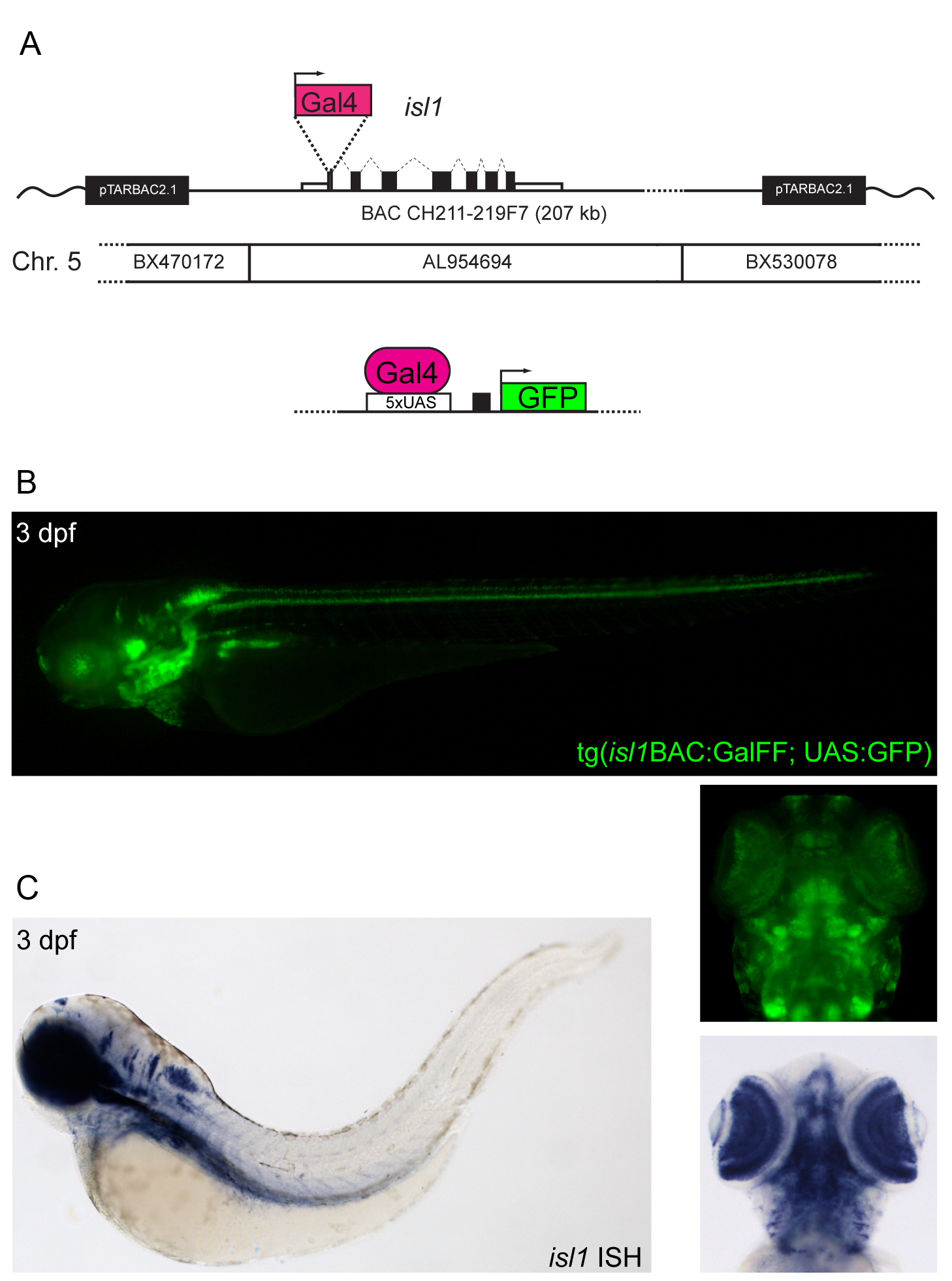

Supplement: Figure S4 — Generation of a reporter transgenic line for isl1. (A) An expression cassette containing the GalFF gene [42] and kanamycin resistance gene was inserted by recombineering into BAC CH211-219F7 at the ATG site of the 1st exon of the isl1 gene. The site of recombineering is approximately 40 kb inside the BAC sequence, minimizing any risk of loss of isl1 regulatory sequences. The recombined BAC was then injected in a tg(UAS:GFP) background [1] to obtain the fluorescent Isl1 expression reporter line Tg(Isl1BAC:GalFF; UAS:GFP). (B) GFP expression pattern of the Tg(Isl1BAC:GalFF; UAS:GFP) line at 3 dpf. (C) Isl1 ISH on WT embryo at 3 dpf. The expression pattern of GFP, reporting for isl1 expression, in the Tg(isl1BAC:GalFF; UAS:GFP) is validated by comparison with the isl1 ISH. Especially visible are the identical expression pattern in the eyes and hindbrain. (TIF) [file pone.0047644.s004.tif]

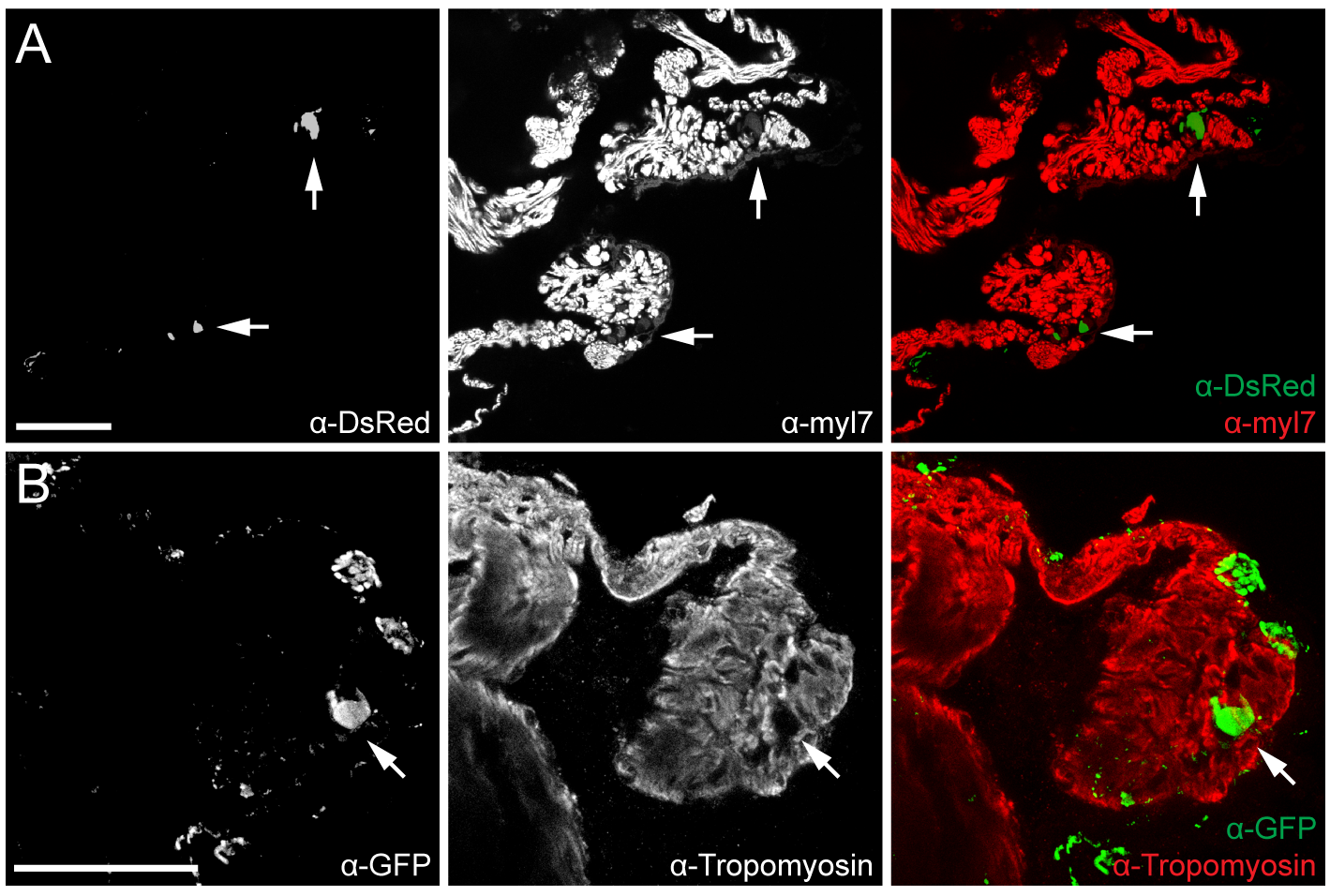

Supplement: Figure S5 — Isl1BAC reporter activity in adult heart. Confocal images of Tg(isl1BAC:GalFF; UAS:RFP; myl7:eGFP) after immunolabeling with anti-RFP and anti-GFP antibodies (A), or Tg(isl1BAC:GalFF; UAS:GFP) after immunolabeling with anti-GFP and antitropomyosin antibodies (B). Isl1 expressing cells (indicated with arrows) are located at the base of the venous valves and contain much lower levels of myosin light chain or tropomyosin compared to surrounding myocardial cells. Axonal Isl1/GFP+structures are visible at the outer surface of the myocardium. Scale bars represent 50 µm (TIF) [file pone.0047644.s005.tif]
